# Supplementary material for: Acceptability of Clinical Trials on COVID-19 during Pregnancy among Pregnant Women and Healthcare Providers: A Qualitative Study
Source: Int J Environ Res Public Health. 2021 Oct 13;18(20):10717. doi: 10.3390/ijerph182010717 (PMC8535397; doi:10.3390/ijerph182010717)
Supplement: Supplementary file 1 [file ijerph-18-10717-s001.zip › Supplementary file 1_interview guide.pdf]

_ Interview number: _ _ _ - _ _ Means of data collection: ________________ Day: _ _ / _ _ / _ _ Hour: _ _ / _ _

SSI question guide: AcCOV study

TOPIC	INTERVIEW QUESTIONS<br>(in black: only for pregnant women, in blue: for pregnant women and healthcare professionals, *in green: only for healthcare professionals*)	COMMUNICATION ASPECTS AND INTERVIEWER NOTES
**Personal overview**	-studies, academic training / job<br>-supports received during pregnancy<br>-life conditions (everyday life/how she feels/other pathologies) during pregnancy	
**Knowledge about COVID-19**	-what do you know about COVID-19?<br>-symptoms?<br>-how does it affect pregnant women?<br>-who can get infected?<br>-how is it transmitted?<br>-how to prevent it?<br>-are there treatments against COVID-19?<br>-If this is the case, for whom is its use recommended?<br>-Do you know if they can be used during pregnancy?<br>-How do you get informed about COVID-19 / how did you get this information? when?<br>-Have you been told about some specific measures to take to avoid infection during pregnancy? who?	
**Experiences related to COVID-19**	-Have you had symptoms of COVID-18?<br>-has it been confirmed / diagnosed?<br>-Have you had close contact with a case?<br>-where? (at home? at work? ...)<br>-Did he/she Required hospitalization?<br>-Did you receive any treatment?	

	-Have you been afraid of being infected? and in the future? * *For healthcare professionals...*<br>-Have you been worried about the progress of your pregnancy or the health of your baby?<br>-Do you think that the confinement and the state of alarm has influenced your emotional well-being?<br>-How do you feel now compared with the beginning of the pandemic?<br><br>**--Have you had contact with infected pregnant women?*<br>*-How have they handled the pregnancy? physically and mentally?*	
**Participation in a clinical trial**	--Do you know what a clinical trial is?<br>-Would you participate in a clinical trial to evaluate the effectiveness / safety of a drug? why?<br>-And during pregnancy? why?<br>-And in the specific context of the COVID-19 pandemic? why?<br>-And if we tell you that the drug is hydroxychloroquine? why?<br>-if it is another drug? remdesivir? dexamethasone?<br>-Under what circumstances would you agree to participate? (Probes: *only in the first trimester of pregnancy, only in the last, if there is a much more serious second wave, if it is shown that there is transmission from the mother to the baby, if it is shown that pregnant women are more likely to be infected, if there is evidence that the drug is safe for the baby ...)*<br>-And during breastfeeding? why?<br>[**In the case of healthcare professionals, include:*<br>-would you recommend your patients to participate in a clinical trial?<br>-And during pregnancy?]<br>-If there is another COVID-19 outbreak now or later, similar to the one in March-April, would you change any of your answers?	
